# Space-Time Point Pattern Analysis of Flavescence Dorée Epidemic in a Grapevine Field: Disease Progression and Recovery

**DOI:** 10.3389/fpls.2016.01987

**Published:** 2017-01-06

**Authors:** Federico Maggi, Domenico Bosco, Luciana Galetto, Sabrina Palmano, Cristina Marzachì

**Affiliations:** ^1^School of Civil Engineering, The University of Sydney, SydneyNSW, Australia; ^2^Istituto per la Protezione Sostenibile delle Piante, Consiglio Nazionale delle RicercheTurin, Italy; ^3^Dipartimento di Scienze Agrarie, Forestali e Alimentari, Entomologia, Università degli Studi di TorinoTurin, Italy

**Keywords:** flavescence dorée, space-time epidemics, randomness, pattern, *Scaphoideus titanus*, *Vitis vinifera*

## Abstract

Analyses of space-time statistical features of a flavescence dorée (FD) epidemic in *Vitis vinifera* plants are presented. FD spread was surveyed from 2011 to 2015 in a vineyard of 17,500 m^2^ surface area in the Piemonte region, Italy; count and position of symptomatic plants were used to test the hypothesis of epidemic Complete Spatial Randomness and isotropicity in the space-time static (year-by-year) point pattern measure. Space-time dynamic (year-to-year) point pattern analyses were applied to newly infected and recovered plants to highlight statistics of FD progression and regression over time. Results highlighted point patterns ranging from disperse (at small scales) to aggregated (at large scales) over the years, suggesting that the FD epidemic is characterized by multiscale properties that may depend on infection incidence, vector population, and flight behavior. Dynamic analyses showed moderate preferential progression and regression along rows. Nearly uniform distributions of direction and negative exponential distributions of distance of newly symptomatic and recovered plants relative to existing symptomatic plants highlighted features of vector mobility similar to Brownian motion. These evidences indicate that space-time epidemics modeling should include environmental setting (e.g., vineyard geometry and topography) to capture anisotropicity as well as statistical features of vector flight behavior, plant recovery and susceptibility, and plant mortality.

## Introduction

Flavescence dorée (FD) is one of the most important and damaging phytoplasma diseases of grapevine (*Vitis vinifera* L.) causing yellowing or reddening and downward rolling of leaves, drying of inflorescences and bunches, and lack of cane lignification (Caudwell, 1990). The FD phytoplasma is a wall-less bacterium belonging to the 16S ribosomal subgroups V-C and –D (Angelini et al., 2001), with three main genetic clusters based on *map* and *rps*C gene sequences (Martini et al., 2002; Arnaud et al., 2007). FD is transmitted in a persistent and propagative manner by the grapevine feeder *Scaphoideus titanus* Ball (Hemiptera Cicadellidae) (Schvester et al., 1963), which was introduced in Europe from North America in the twentieth century (Vidano, 1964; Bertin et al., 2007; Papura et al., 2012). The vector is a monovoltine leafhopper that overwinters as egg laid under grapevine bark. Infected grapevines usually show symptoms the year after infection but longer incubation periods are possible depending on plant response to the pathogen and environmental factors (Caudwell et al., 1987). Grapevines can recover from FD disease at a rate that depends on the grapevine cultivar (Bellomo et al., 2007; Morone et al., 2007; Belli et al., 2010), but the underlying physiological and molecular mechanisms are not yet fully understood (Caudwell and Larrue, 1986; Musetti et al., 2007; Margaria and Palmano, 2011; Gambino et al., 2013; Vitali et al., 2013). In our earlier studies, we found that *S. titanus* could not acquire FD from asymptomatic and recovered vines (Galetto et al., 2014), and that vector acquisition efficiency was low when feeding on plants with low phytoplasma load (Bressan et al., 2005b; Galetto et al., 2016); in those experiments, it was found that recovered plants were symptomless and returned negative PCR test against FD phytoplasma (Galetto et al., 2014). In some cases, severely affected grapevines may dye; yet, symptomatic plants are normally replaced with healthy plants within the standard viticultural practices. While FD was initially detected in France in the 1950s (Caudwell, 1957), it is now present in several grapevine growing areas of Europe (EFSA-PLH Panel on Plant Health, 2014), including the Piemonte region in north-western Italy (Gotta and Morone, 2001; Marzachì et al., 2001). In this region, disease incidence is locally very high, possibly due to an increasing *S. titanus* population on wild grapes as noticed by the authors and also in Pavan et al. (2012). Over the past decade, milder winters and warmer springs and falls, have provided longer periods for *S. titanus* stages to complete and may account for this population increase (Chuche and Thiery, 2009; Rigamonti et al., 2011). A greater *S. titanus* population is the first factor contributing to a faster spread of FD disease; earlier studies (e.g., Morone et al., 2007; Lessio et al., 2009) show that FD can spread rather rapidly in the absence of *S. titanus* control measures, and can infect the totality of vines within a vineyard in only few years. Insecticide applications have shown that *S. titanus* populations can be reduced in average by about 80–95% (Bosio et al., 2001; Gusberti et al., 2008), but predictive modeling shows that full FD suppression is not likely with insecticides alone in the short term, and that roguing should be implemented in conjunction with insecticide applications (Maggi et al., 2014a,b; Lessio et al., 2015). In spite of the remarkable resources devoted to control *S. titanus* presence with mandatory insecticide applications and compensate yield losses (more than €34 million in the European Union), the vector is expanding its presence in western, eastern, and southern Europe (Chuche and Thiery, 2014; Digiaro et al., 2014).

The role played by *S. titanus* on FD spread depends on its life stages including hatching, molting and emerging rates as well as their duration, aging, and survival to predation and parasitism. FD affects *S. titanus* fitness (Bressan et al., 2005a), and this can therefore introduce nonlinear feedbacks on its reproduction, and movement in space and over time, hence on FD spread. The complex features of the FD epidemics, including acquisition and transmission, latency in vector insect and host plant, and fitness effects on vector and host, have been explored by mathematical descriptions of FD dynamics and epidemiology in various recent works (Rigamonti et al., 2011, 2013; Maggi et al., 2013, 2014a,b; Lessio et al., 2015) and have provided indicative explanations of the large temporal heterogeneity in FD outbreaks not only over regional scales but also from site to site in the same region. Earlier analyses have highlighted the relatively sparse nature of *S. titanus* after aggregated counting at intra and regional scales (Linder and Jermini, 2007; Seljak, 2008). At smaller scales, ranging from the plant to the field, aggregation of *S. titanus* nymphs (Lessio and Alma, 2006; Chuche et al., 2011) and adults (Bosco et al., 1997) may explain the presence of source points of FD outbreak, which may reflect the apparently sparse spatial distribution at larger scales. On the one hand, low incidence of aggregated infection points with sparse distribution may be related to primary infections, which typically occur with evident boundary infections due to incoming infected leafhoppers. On the other hand, high incidence and aggregated distributions of infection points may be sign of secondary infections, which are characterized by aggregated patterns caused by infected vectors that acquired and transmit the phytoplasmas within the vineyard. These different patterns seem to be persistently found in other insect-borne plant diseases including the potato Zebra Chip disease (Henne et al., 2012), Citrus Huanglongbing (Gottwald, 2010), Verticillium Wilt in mint (Johnson et al., 2006), *Passalora fulva* in tomato (Kawaguchi and Suenaga-Kanetani, 2014) and others, but also underline that the degree of aggregation of insect vectors and infected plants may vary with the sampling scale. This feature, therefore, suggests that no self-similarity is present in the spatial distribution of epidemics and that the way the degree of randomness is measured may be biased by the method itself, or that, alternatively, the degree of randomness is an intrinsically varying quantity in insect-borne plant diseases (e.g., Cressie, 1993). This opens a number of questions on whether methods to measure the degree of randomness should be implemented at multiple scales, and whether various methods should be applied. Although there is no epidemiological mapping over regional-to-continental areas of the FD progression that uses symptoms observed in individual plants, data exists over the field scale (e.g., Morone et al., 2007; Pavan et al., 2012; Rigamonti et al., 2013). It is therefore possible to retrieve space-time point pattern statistics that may highlight advances in heterogeneity of FD epidemics and trends in its progression and regression using diverse methods. Advances in measuring statistical properties of spatial epidemics (Cressie, 1993; Diggle, 2003; Lawson, 2006; Brauer and Castillo-Chavez, 2012), allow us to elaborate to a greater detail the features of space and time of FD epidemic dynamics along those lines; the information retrieved in this way can be used to include preferential directions in space-time dynamic models of epidemic spread, the plant response to infection such as mortality or recovery, or include characteristics of the flight behavior of the vector.

This work reports results of space-time point pattern analyses of an FD epidemic occurring in an experimental vineyard located in Cocconato, Piemonte region, Italy, and therefore it illustrates the spatial distribution and incidence of infected vines within the vineyard. The Piemonte region is a major contributor to the economic value of grape production and wine processing for the domestic and export market and, along with the historical oenology tradition, viticulture in Piemonte is a strategic asset with important economic and social implications. The analyses focused on both static (year-by-year) and dynamic (year-to-year) epidemic patterns measured by the Complete Spatial Randomness (CSR) approach and tested by the quadrat and nearest-neighbor methods. The former was carried out over multiple scales to test whether CSR was stationary. Epidemic isotropicity was measured by means of static bidirectional and multidirectional two-dimensional correlation analyses. Emphasis was also given to the statistical characteristics of the space-time dynamic distributions of newly symptomatic and recovered plants to highlight similarities and differences in FD progression and regression against the overall epidemic characteristics.

## Materials and Methods

### Experimental Vineyard and FD Measurements

**FIGURE 1:**
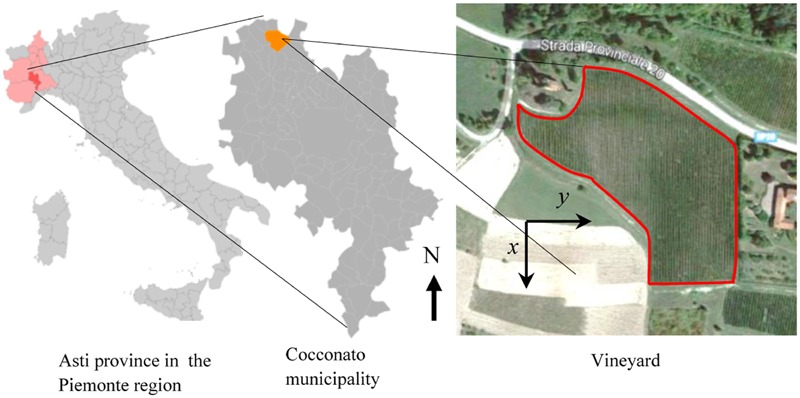
**Geographic location of the experimental Barbera vineyard in the Cocconato municipality, Asti province, in the Piemonte region, Italy.** The right panels show the geographical north (N) and the system of reference used for analytical computations. Satellite image from Google.

Flavescence dorée is present in Italy since the early seventies (Belli et al., 1973), and systematic surveys of infected plants and monitoring of vector presence have been conducted since the late nineties in various sites in Piemonte, Italy. The vineyard of interest in this work is located in the Cocconato municipality, province of Asti (45°04′58.4′′N 8°03′21.1′′E, N-S orientation), and it extends over a surface area of about 1.75 ha (17,500 m^2^) cultivated with 8749 Barbera grapevines (**Figure 1**). The vineyard consists of 76 rows of variable number of spur-trained plants (from 21 to 194); rows have an average distance of 2.5 m, while plants along rows have an average distance of 0.8 m. The average plant density in the vineyard is about 5000 ha^-1^ (0.5 m^-2^). The vineyard has been under conventional agricultural practices, and has been sprayed with thiamethoxam insecticide in the second half of June to control *S. titanus* nymphs, and with Chlorpyriphos-ethyl 1 month later to control adults, as per the schedules enforced by the Regional Phytosanitary Service. For the purpose of this experiment, infected (symptomatic) grapevines have not been uprooted; symptomatic plants have been pruned during the summer.

All plants in the vineyard have been monitored for FD symptoms between July and mid-August from 2011 to 2015, and data are still collected yearly to continue monitoring. Each vine was visually inspected and the simultaneous presence of three FD-specific symptoms was used as a criterion to map FD symptomatic vines in space and time. At least 10 FD-symptomatic and healthy vines were randomly sampled every year to validate FD presence by PCR with universal phytoplasma primers followed by nested PCR with 16SrV-specific primers according to Morone et al. (2007). Likewise, dead and removed vines were mapped in space and time. These procedures resulted in space-time point pattern survey maps of symptomatic *M_t_*(*x, y, t*), removed *M_c_*(*x, y, t*), and dead *M_d_*(*x, y, t*) vines as a function of space (*x, y*) and time *t*, with *x* and *y* the coordinates along and across rows in the vineyard, respectively. Labeled vines were identified by 1 s in those maps. Maps of newly symptomatic *M_nt_*(*x, y, t*) and recovered (without symptoms) vines *M_r_*(*x, y, t*) at time *t* were obtained as

(1) $$M_{\text{nt}}(x,y,t)=H[M_{\text{t}}(x,y,t)-M_{\text{t}}(x,y,t-\Delta t)]$$

(2) $$M_{\text{r}}(x,y,t)=H[M_{\text{t}}(x,y,t-\Delta t)-M_{\text{t}}(x,y,t)]$$

with the Heaviside function *H*(*x*) = 1 for *x* ≥ 1 or *H*(*x*) = 0 otherwise. All maps consisted therefore of matrixes of values equal to 1 when the plant at that location (*x, y*) and time *t* was to be marked, or it was equal to 0 otherwise.

### Plant Counting and Incidence

With these maps, counting was carried out for the number of symptomatic *P_t_*(*t*), removed *P_c_*(*t*), dead *P_d_*(*t*), newly infected *P_nt_*(*t*), and recovered plants *P_r_*(*t*) in year *t*. Their incidence was calculated by normalizing these counts by the total number of plants *N* in the vineyard.

### Complete Spatial Randomness (CSR) Tests

Flavescence dorée epidemics maps from space-time point pattern surveys were analyzed to measure the statistical significance of departure from the hypothesis of CSR using the quadrat and nearest-neighbor methods. The two methods are conceptually different, the former being an area-based while the latter being a distance-based method (Gatrell et al., 1996). The advantage provided by the quadrat method is that it allows application at different sampling scales, hence it provides indications about space stationarity of the epidemic statistics, while the advantage of the nearest-neighbor method is that a greater number of statistical quantities can be retrieved and that these are scale-independent as long as the punctual density of infected plants is not exceedingly high (Orensanz et al., 1998).

To test the CSR hypothesis using the quadrat method, a δ-covering of a generic map *M*(*x, y, t*) was used, with quadrats of size δ expressed either in the unit of surface area [*L*^2^] or by means of the number of plants (i.e., dimensionless). The number of symptomatic plants *n_i_* were counted in each quadrat δ*_i_*, and the mean 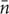 and variance *s*^2^ of non-zero counts were calculated. Assuming that the Poisson quadrat count approximation can be used as a proxy to the binomial distribution of infected plants (Cressie, 1993; Diggle, 2003), the coefficient of dispersion D = s^2^/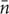 should be *D* = 1 for point patterns that are completely randomly distributed (i.e., in the Poisson sense). Values *D* < 1 indicate that the variance in quadrat counts is small and suggest that symptomatic plants are rather dispersed (more than in a complete random distribution); values *D* > 1 indicate that the variance in quadrat counts is large and symptomatic plants may be clustered or aggregated (more than in a complete random distribution). Using a tolerance of ±0.1, values *D* = 1 ± 0.1 may not reject the CSR hypothesis, but greater variations suggest that the CSR hypothesis can be rejected. Because counts may depend on the quadrat of the δ-covering, the quadrat method was applied for various δ-values ranging from 2 × 2 plants up to 24 × 24 plants (along row × across row) that is equivalent to surface areas ranging between 8 and 1152 m^2^. The method was applied to the surveys from 2011 to 2015 to highlight whether the CSR hypothesis was true and if its validity depended on the δ-covering size and time, the latter being also an expression of CSR validity against FD incidence in the vineyard.

To test the CSR hypothesis using the nearest-neighbor method, the minimum Euclidian distance *d_ij_* between any two independent points with coordinates (*x_i_, y_i_*) and (*x_j_, y_j_*) was calculated. Because the total number of symptomatic plants in the vineyard in a given year *t* is *P_t_*(*t*) and independent couples of points were used, the sample size was *m* = *P_t_*(*t*)/2. When *P_t_*(*t*) was uneven, the last point left over after coupling was neglected and removed from the sample. Next, the average 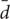 of these independent distances *d_ij_* was calculated and was used to determine the standard normal variable z = (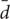 - μ)/σ, with population mean μ = 1/2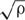 in [1/L] and standard deviation 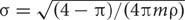 in [1/L], where ρ = m/S was used for the average density in [1/L^2^] of symptomatic neighboring plants in the vineyard (Cressie, 1993; Diggle, 2003). If the CSR hypothesis is true, *z* should be a sample extracted from a normal distribution with mean equal to zero and standard deviation equal to one. Given the level of significance a = 0.05, the CSR hypothesis can be rejected in the two-tail test if |z| > z_α/2_ (i.e., |z| > z_α/2_ > 1). In this criterion, a tolerance ±0.1 was used. As for the quadrat method, this test was applied to all surveys from 2011 to 2015. An indication of whether the CSR hypothesis could be rejected was also provided by comparing 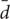 and μ; values 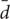 < μ indicate that, for the given density ρ in the vineyard, symptomatic plants are in average too close to their nearest neighbor symptomatic plant to be randomly distributed, thus suggesting that infections are clustered. In contrast, values 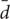 > μ indicate that symptomatic plants are more dispersed than when these are randomly distributed.

A number of indexes exist to provide an additional measure of clustering including the index of cluster size (David and Moore, 1954), cluster frequency (Douglas, 1975), and patchiness (Morisita, 1959; Lloyd, 1967), which were not used in these analyses as they base on analogous combinations of average and standard deviation as *D*.

### Two-Dimensional Correlation Function

A measure of the isotropicity (symmetry and eccentricity) of point patterns in FD epidemic in the study site was achieved by using a two-dimensional multidirectional correlation function. Given the generic discrete map *M*(*x, y, t*) as per the Section “Experimental Vineyard and FD Measurements,” the autocorrelation function Γ*_M_* is calculated as (Sethna, 2006).

(3) $$\Gamma_{\text{M}}(x,y,t)\frac{1}{N}\sum_{\tau_{\text{x}}}\sum_{\tau_{\text{y}}}M(x+\tau_{\text{x}},y+\tau_{\text{y}},t)\cdot M(x,y,t)$$

with τ*_x_* and τ*_y_* the spatial distance and *N* the total number of marked plants in map *M*(*x, y, t*) in year *t*. Note that Eq. 3 becomes Γ_M_(x,y,t)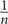 ∑_τ_x__ ∑ _τ_y__ ⋅ [M(x,y,t)]^2^ = 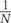 ⋅ N = 1 for τ*_x_* = 0 and τ*_y_* = 0, while any values τ*_x_* ≠ 0 and τ*_y_* ≠ 0 lead to Γ_M_(x,y,t) < 1. The autocorrelogram represents the rate at which the correlation Γ*_M_*(*x, y, t*) with a point pattern at a position identified by the vector (τ*_x_*, τ*_y_*) is lost. For this reason, Γ is also associated to a memory effect of the underlying process that emphasizes the length scale at which any effects of processes within (τ*_x_*, τ*_y_*) surroundings are lost (Tennekes and Lumley, 1972). In this work, Eq. 3 was calculated relative to the surveys from 2011 to 2015 and was used to determine the year-by-year (static) isotropicity of symptomatic plants in maps *M_t_*(*x, y, t*). Isotropicity can be highlighted by isopleth lines, and the isotropicity eccentricity can be calculated to measure the degree of symmetry of year-by-year epidemic progression.

Because the cultivation setting in the vineyard is itself strongly anisotropic as plants are aligned along regular rows, the bidirectional autocorrelation functions Γ*_M, y_*(*x, t*) and Γ*_M,x_*(*y, t*) for the generic map *M*(*x, y, t*) were calculated by alternatively varying only one of either τ*_x_* or τ*_y_*, and fixing the other to zero; this procedure corresponded to taking cross-sections of Γ*_M_*(*x, y, t*) at τ*_x_* = 0 and τ*_y_ =* 0 along the *y* and *x* directions, respectively. Comparison of the Γ*_M, x_*(*y, t*) and Γ*_M, y_*(*x, t*) across and along rows, respectively, provides evidence that correlation between point patterns (memory) may be lost more rapidly in one direction rather than the other. The correlation function can also be applied to two different maps in a similar way as in Eq. 3, thus resulting in the cross-correlation function.

### Point Patterns Analyses of Epidemic Progression and Regression

The analytical methods described in the Section “Two-Dimensional Correlation Function” and applied to space-time static (year-by-year) point patterns can also be used to identify space-time dynamic features (year-to-year) of epidemic progression and regression when applied to maps of newly symptomatic *M_nt_*(*x, y, t*) and recovered *M_r_*(*x, y, t*) plants. When compared to static analyses, dynamic analyses can quantify whether the statistics of the epidemic dynamics will depart from static ones over a sequence of time-correlated measurements, thus providing indications on whether epidemic progression and regression are stationary or change over time.

In addition to point pattern progression and regression investigated by means of the correlation function Γ of Eq. 3, the probability distribution of distance *d* and direction 𝜃 between newly symptomatic and recovered plants in year *t* relative to the nearest symptomatic plants in the previous year *t* - Δ*t* were carried out. This analysis was aimed at comparing similarities in the progression (newly symptomatic) and regression (recovered) of FD epidemic.

## Results

Selected results of a comprehensive space-time analysis are presented in the following sections.

### Epidemiological Maps, Point Counting, and Incidence

**FIGURE 2:**
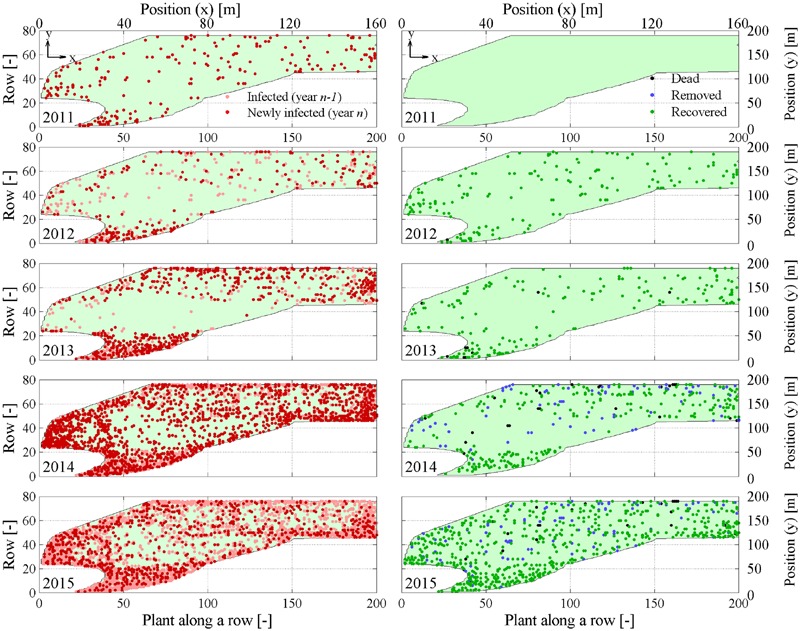
**Yearly maps of symptomatic *M_t_*(*x, y, f*) and newly symptomatic *M_nt_*(*x, y, f*) plants (left column), and *dead Mj*(*x, y, t*), removed *M_c_*(*x, y, t*), and recovered *M_r_*(*x, y, t*) plants (right column).** Maps are relative to years 2011–2015 during flavescence dorée (FD) epidemic in the experimental site.

**FIGURE 3:**
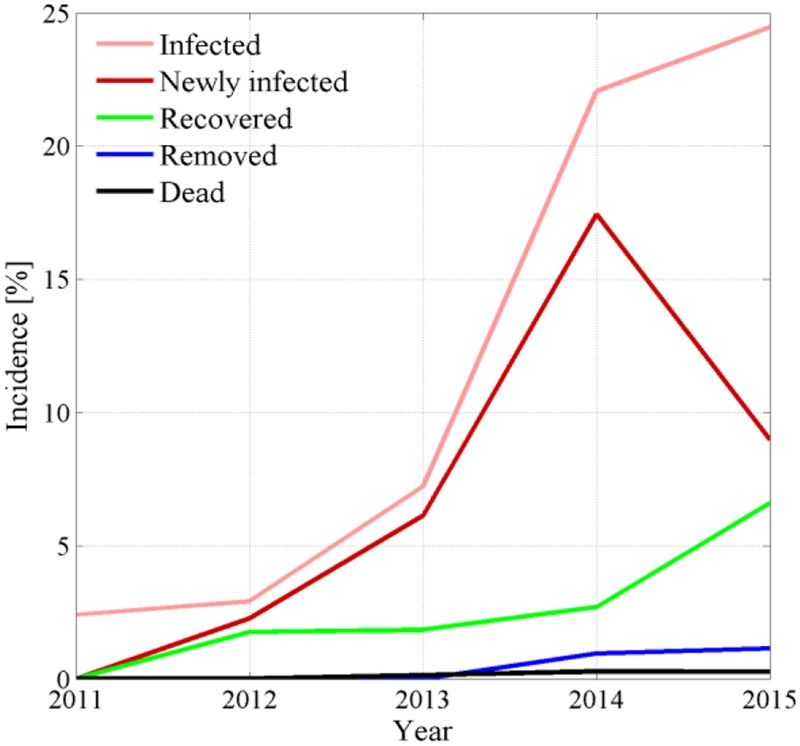
**Yearly incidence of symptomatic, newly symptomatic, recovered, removed, and dead plants recorded during FD epidemic in the experimental site**.

Space-time point pattern maps show progression of FD symptomatic plants every year and emphasize newly infected plants not showing symptoms the previous year (**Figure 2**, left column). Maps also depict dead, removed, and recovered plants in each year (**Figure 2**, right column). Absolute counts *P_t_*(*t*), *P_d_*(*t*), *P_r_*(*t*), and their incidence were represented over time in **Figure 3**.

Eye inspection of maps and incidence show that the vineyard was initially exposed to an apparently sparse spatial infection distribution, but also show a rapid epidemic progression from the upper (south-eastern) and lower (north-western) boundaries to reach an incidence *P_t_*(*t*)/*N* of about 25% in only 3–4 years. Years 2013 and 2014 showed a dense apparent aggregation of symptomatic plants along rows near the upper and lower boundaries (**Figure 2**), suggesting that the infection was brought into the vineyard from *S. titanus* vectors entering the vineyard from those sides. Predominant *S. titanus* migration probably occurred from the south-western side where there is no physical barrier and, to a lower extent, the eastern side, where a low-plant fencing is present (western direction, **Figure 1**, left column); less migration appeared through the northern boundary, where a regional road marks the watershed divide.

Flavescence dorée spread initiated before 2011 in that vineyard, but maps show that plant mortality did not occur until 2013 (black dots, **Figure 2**, right column), suggesting that infected plants may survive up to 3 or more years before dying. Note that mortality, however, only reached less than 0.05% incidence over the total number of plants. Analysis of point pattern in individual plant mortality is presented later to highlight statistics of average grapevine survival to FD infection.

Plant recovery (**Figure 2**, right column, green dots) was found to be more relevant than mortality, with an incidence that reached about 7% of the total plant population in 2015 (**Figure 3**), and about 30% of plants with symptoms in the previous year. Plant recovery in this vineyard was similar to that observed in earlier analysis in Morone et al. (2007) relative to Barbera, Bonarda, and Cortese vineyards. The yearly recovery rate estimated from these maps was calculated after Maggi et al. (2014a,b) as r = -[P_t_(t) - P_t_(t - Δt)]/[Δτ ⋅ P_t_(t - Δt)], with Δ*t* = 1 year, and ranged between 1.37 × 10^-5^ 1/day in year 2013 and 1.73 × 10^-3^ 1/day in year 2012, with an average over all years of 5.73 × 10^-4^ 1/days (equivalent to 0.21 1/year). This figure meets the recovery rate estimated from mechanistic modeling of various FD epidemics in Maggi et al. (2014b) against observations in four vineyards cultivated with Cortese, Dolcetto, Bonarda and Barbera. Additionally, this recovery rate suggests that a plant with FD symptoms recovers, in average, after about 4–5 years, although the chance that a symptomatic plant recovers in a period of 5 years was about 25% to 35% (**Figure 3**).

### Point Pattern Randomness (CSR Hypothesis)

Maps of symptomatic plants (**Figure 2**, left column) were analyzed with the quadrat and nearest-neighbor methods to test the CSR hypothesis as detailed in the Section “Complete Spatial Randomness (CSR) Tests.”

**FIGURE 4:**
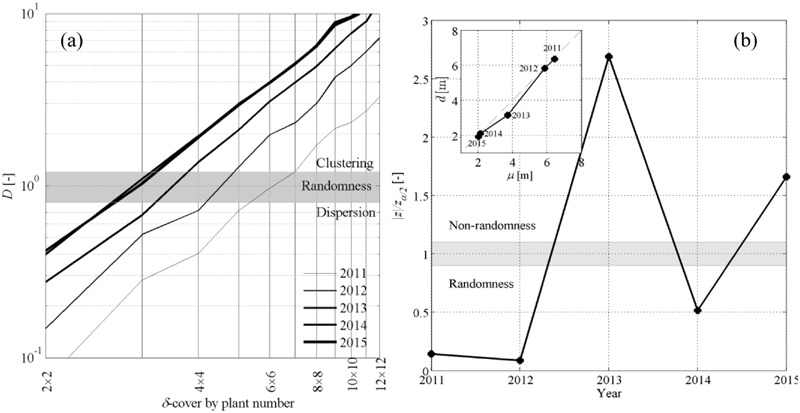
**(a)** Dispersion coefficient *D* as a function of the δ-covering size in the quadrat method. **(b)** *Z*-test applied to the nearest-neighbor method represented against time, with inset representing the average sample distance 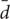 between nearest neighbors against the population average μ.

Calculation of the dispersion coefficient *D* with the quadrat method showed that point patterns were randomly distributed in space (i.e., *D* ≈ 1 ± 0.1) only at relatively small scales, i.e., for δ-covering ranging between 3 × 3 and 6 × 6 plants. Dispersion was *D* < 1 for smaller δ values and was *D* > 1 for greater δ values. The CSR hypothesis was therefore likely satisfied only over a narrow band in spatial scales. The double logarithmic representation in **Figure 4a** also suggests that the rate at which spatial clustering became evident relative to coarsening of the δ-cover had an exponential trend, suggesting that the dominant feature in the test vineyard was the aggregated, clustered nature of symptomatic plants. Analysis of *D* values over time also showed that clustering became more and more important as the epidemic progressed over time (thin against thick lines, **Figure 4a**) and the incidence of symptomatic plants increased. Clustering can be interpreted as the result of new infections occurring relatively close to existing symptomatic plants, and may be linked either to secondary infections caused by *S. titanus* individuals already present in the field or to a limited movement of the incoming infected leafhopper between adjacent plants. Overall, the quadrat method suggests that the CSR hypothesis can be rejected and that point patterns were mostly clustered.

Nearest neighbor statistics showed that the CSR hypothesis could not be rejected in year 2011 and 2012, while the hypothesis was weaker in 2014 (**Figure 4b**). This meets with the comparison between the sample and population average distance 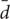 and μ (**Figure 4b**, inset), where 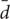 slightly departed from μ in 2013 and 2015. Rejecting the CSR hypothesis through the *z*-test has the implication that the space point patterns do not follow a Poisson distribution, but this does not allow us to make any statement on whether point patterns are aggregated or dispersed. This information was rather retrieved from 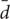 and μ; when point patterns could not be assumed to be randomly distributed (years 2013, 2015), condition 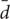 < μ indicated that nearest-neighbor symptomatic plants were mostly aggregated.

### Point Pattern Isotropicity

**FIGURE 5:**
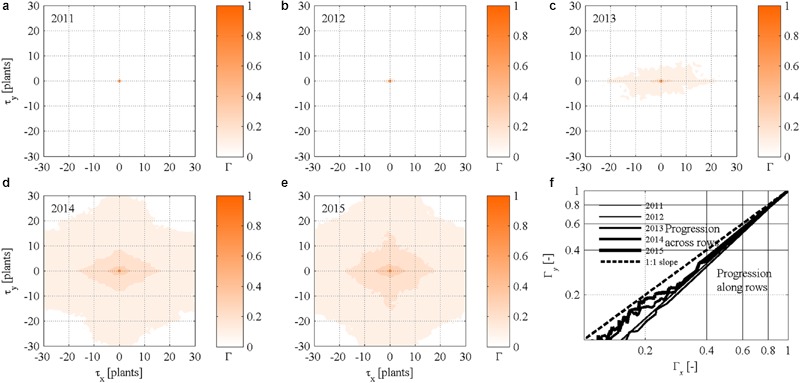
**(a–e)** Static (year-by-year) two-dimensional autocorrelograms Γ_Mt_(x,y,t) of FD symptomatic plants in the experimental site. **(f)** Scatter plot of bidirectional two-dimensional autocorrelation Γ_M,x_(y,t) and Γ_M,y_(x,t) of FD symptomatic plants calculated for τ*_y_ =* 0 and τ*_x_* = 0, respectively.

Autocorrelograms of the static (year-by-year) autocorrelation functions Γ*_M_*(*x, y, t*) calculated for maps *M_t_*(*x, y, t*) of symptomatic plants show substantially no correlation between spatial points in year 2011 and 2012 (**Figures 5a,b**), with an exception at the spatial distance |(τ*_x_*, τ*_y_*)| = 0 that corresponded to Γ*_M_*(0, 0, *t*) = 1. From year 2013, the autocorrelograms show a substantial increase in correlation also for distances |(τ*_x_*, τ*_y_*)| > 0, which were mainly caused by an increasing incidence of symptomatic plants (**Figures 5c–e**). Note, however, that correlations were very weak, and the influence of symptomatic plants on the surroundings did not exceed a distance of about | (τ*_x_*, τ*_y_*)| = 2–4 plants (i.e., 1.5–3 m).

Autocorrelograms also suggest that the increasing number of symptomatic plants progressed year-by-year in a non-isotropic way especially in year 2013 and 2014 as indicated by elongated isopleths (**Figures 5c,d**). Correlation increased in 2015 but anisotropicity faded out, possibly due to the high incidence of symptomatic plants (see also **Figure 2**, left column). Because eccentricity of isopleths occurred along rows in the *x* direction, and it was particularly visible in year 2013 and 2014, it was inferred that epidemic spread more rapidly along than across rows. This result meets the bidirectional autocorrelation functions Γ_M_t_,x_(y,t) and Γ_M_t_,y_(x,t) represented against each other in **Figure 5f**, with the curves located below the 1:1 line in all years.

### Analysis of Newly Symptomatic Plants

**FIGURE 6:**
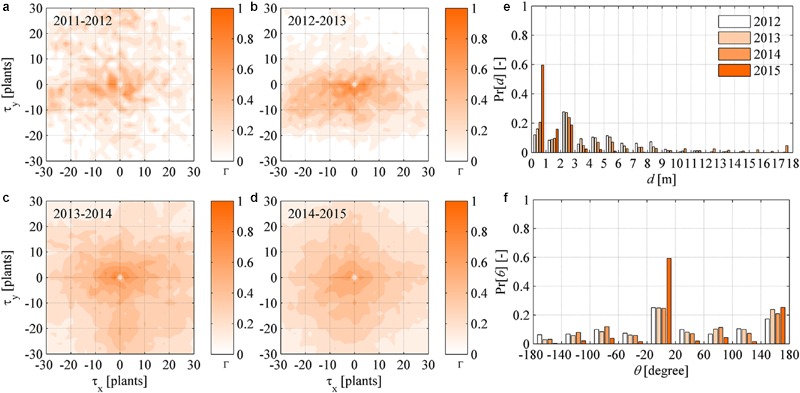
**(a–d)** Dynamic (year-to-year) two-dimensional crosscorrelograms of FD newly symptomatic plants in year *t* relative to symptomatic plants in year (*t* - Δ*t*). **(e–f)** Yearly probability distributions of the distance *d* and direction 𝜃 between newly symptomatic plants in year *t* and symptomatic plants in year (*t* - Δ*t*), with 𝜃 measured in degrees relative to the direction 𝜃 = 0 of rows in the experimental site.

The cross-correlation function was calculated for the maps of symptomatic plants *M_t_*(*x, y, t* - Δ*t*) in year (*t* - Δ*t*) and newly symptomatic plants *M_nt_*(*x, y, t*) in year *t*, with the purpose to highlight the dynamic (year-to-year) spatial correlation existing between infection sources and progression of infection at two succeeding years. The cross-correlograms in **Figures 6a–d** show that a correlation existed at spatial scales that extended more widely than those appearing in the static (year-by-year) point pattern autocorrelations of symptomatic plants in **Figure 5**. However, noise in these correlations suggest that there was no clear pattern if not the preferential direction of infection along rows already observed in the Section “Point Pattern Isotropicity.” A possible explanation for this is the statistical feature of *S. titanus* vector flight, which may be randomly distributed with a preference along rows, as evidenced in earlier studies (Lessio et al., 2009).

To highlight the aspect mentioned above, the probability distribution of the distance *d* and direction angle 𝜃 between any newly symptomatic plants in a year and their nearest symptomatic plant in the previous year were determined for all years. The probability distributions of *d* followed an irregular negative exponential function with accumulation toward small *d* values and a long tail over large *d* values in all years (**Figure 6e**). These irregular exponential distributions are considered to be a reflection of point patterns in symptomatic plants not obeying complete randomness as per the CSR tests carried out in the Section “Point Pattern Randomness (CSR Hypothesis)” – if the CSR hypothesis could not be rejected, the probability distributions of *d* should rather follow a negative exponential (Poisson) function. Overall, from 2012 at least 50% of newly symptomatic plants were found within 3 m distance from plants already symptomatic. The probability distribution of 𝜃 did not follow any theoretical distribution at a first sight (**Figure 6f**), but it was possible to recognize a nearly uniform probability over angles ranging between -180 and 180°, with the exception of the angle of direction of rows along the *x* coordinate (about 0 and 180°). Finally, it was noted that those trends were accentuated as FD incidence increased, that is, the likelihood that newly symptomatic plants occurred nearby already symptomatic plants increased year after year to 90% of occurrences within 3 m (**Figure 6e**, yellow bars) and it increased to more than 80% of occurrences in the direction of rows (**Figure 6f**, yellow bars).

### Analysis of Recovered Plants

**FIGURE 7:**
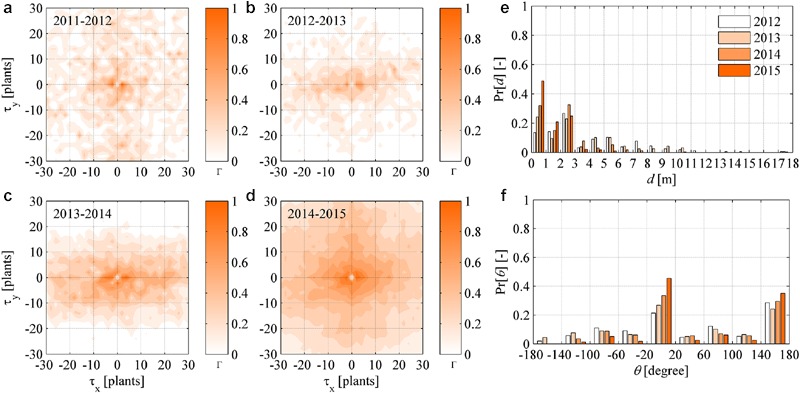
**(a–d)** Dynamic (year-to-year) two-dimensional crosscorrelograms of FD recovered plants in year *t* relative to symptomatic plants in year (*t* - Δ*t*). **(e–f)** Yearly probability distributions of the distance *d* and direction 𝜃 between newly symptomatic plants in year *t* and symptomatic plants in year (*t* - Δ*t*), with 𝜃 measured in degrees relative to the direction 𝜃 = 0 of rows in the experimental site.

The spatial pattern in plant recovery was investigated in a similar way as in the Section “Analysis of Newly Symptomatic Plants.” Given the maps of symptomatic plants *M_t_*(*x, y, t* - Δ*t*) in year (*t* - Δ*t*) and recovered plants *M_r_*(*x, y, t*) in year *t*, the cross-correlation functions showed a weak strength in years 2012–2014, and a relative increase in 2015 (**Figures 7a–d**). Notably, correlation in 2015 did not show noisy levels as those in **Figures 6a–d**. Overall, there was no clear correlation between the location of plants that recovered and those showing symptoms in the previous year, suggesting that plant recovery was not significantly influenced by nearby infections. Indeed, plant recovery is driven by a defense mechanism of the infected plant itself (Musetti et al., 2007; Gambino et al., 2013; Santi et al., 2013) that has no relations with the sanitary status of neighboring plants. Tracking recovered plants against newly infected plants in the following years, demonstrated that recovered plants did not reinfect over the time scale analyzed here.

The probability distribution of distance *d* and direction 𝜃 between any recovered plants in a year and their nearest symptomatic plant in the previous year were determined for all years. Also in this case, *d* values were irregularly distributed along a negative exponential distribution, while 𝜃 values showed a nearly uniform distribution with peaks at 0 and 180° (**Figures 7e,f**). Given the results presented here, it was inferred that recovery occurred as an underlying stochastic process not conditioned by the health status of neighboring plants, and that recovery did not show substantial correlation in space.

## Discussion

The set of data for the FD epidemic presented and analyzed here is particularly important because of the density of measurements (individual plants), the tracking of removed, dead, and recovered plants in addition to symptomatic plants, and for the time extent these measurements have been taken for. A number of space-time point pattern analyses over multiple years have elucidated statistical features in FD progression and regression in the field under observation, and key statistical features are discussed in greater details below.

The first characteristic is that space-time point patterns of infected plants were rarely found to satisfy the CSR hypothesis in the Poisson sense except for some length scales in the quadrat test and some years in the nearest-neighbor test. Rather, the two tests seemed to converge to identify point patterns that generally were either sparse islands or aggregated infection clusters, as observed for another leafhopper-transmitted phytoplasma (Beanland et al., 2005). If the hypothesis that primary infections are identified by sparse source points and secondary infections by clustered source points, then the analyses presented here indicate that transition from primary to secondary infection was rather rapid even if these coexisted at some time of epidemic progression and in some areas near the boundaries, where primary infections are generally likely. Note that clustering may be due not only to secondary infections within the vineyard, but also to incoming infected leafhoppers (primary infection) that infect plants close to each other. Note also that distributions of infection source and vector incidence at the boundary were not investigated in this work, but a detailed analysis of migration through boundaries of treated and untreated vineyard showed that *S. titanus* can colonized treated vineyards by migrating after insecticide applications from untreated cultivated vineyards as well as from abandoned vineyards or from wild grapevines surrounding cultivated fields (Pavan et al., 2012).

Our tests suggest that the length scale of sampling is as important a parameter in the CSR validation as the time at which assessment is carried out. Point patterns are therefore intrinsically space and time dependent, and suggest that no self-similarity can be recognized in the space-time domain analyzed here. We recognize, however, that a different interpretation of self-similarity appearance may result from point density counting in the δ-covering used for the quadrat method over different scales such as, for example, over a field and over a region. A richer description of the epidemic evolution, and its progression and regression, may rather be achieved in the framework of multiscale processes, where statistical characteristics of epidemic point patterns may be better understood to occur with different features at different scales in time and space, and brought to light in this work relative length scales ranging from the plant to the field, and over 5 years of observations.

Second, both static and dynamic correlation analyses have persistently shown that symptomatic, newly symptomatic, and recovered plants occur with spatial anisotropicity favoring the direction of rows during the exponential growth phase of epidemic incidence, while anisotropicity faded out when incidence was too high, say from 2014. Anisotropicity always occurred along the *x* coordinate, that is, along vineyard rows, a pattern that is consistent with the vector movement observed also in Lessio et al. (2014). Note, however, that other anisotropic effects related to intrusion of infectious vectors from the boundaries could be captured by eye in the maps of **Figure 2**, which are in line with earlier observations by Pavan et al. (2012). Additional factors affecting epidemic spread and its isotropicity are known to be winds with a prevalent direction, land geomorphology that presents physical barriers, and vector-specific mobility behavior. Note that rows themselves may have affected the wind direction, thus a superposition of effects due to row and forced wind direction may have accentuated FD preferential propagation direction. Factors such as temperature and plant phenology are known to have an impact on the development rate of *S. titanus* (Rigamonti et al., 2013). Hence, all these factors may play a role in determining the epidemic isotropicity from field to regional scale, and could be modified in order to minimize the disease spread.

Finally, the probability distribution of distance and direction between newly symptomatic and recovered plants relative to the closest symptomatic plants in the previous year appeared similar to each other, and highlighted a nearly uniform distribution in the direction, with higher probability along rows, and a nearly regular exponential distributed in the distance. The probability peaks in the row directions (0 and 180°) could reflect the average distance covered by the vector during its staying in the vineyard, which only ranged within 2–4 plants (i.e., 1.5–3 m). In fact, *S. titanus* adults are mostly sedentary and spread over short distances as also evidenced in Lessio et al. (2014). A nearly uniform distribution in all other directions suggest that *S. titanus* vector moves choosing a random direction for generally short flights, that is, about 50% of travel distances were within 3 m. This interpretation of vector flight behavior finds similarities with Brownian motion and random walks theories.

## Summary and Conclusion

Analyses presented here for the characterization of FD epidemic point patterns over a time frame of 5 years using area- and distance-based methods, as well as correlation functions, highlighted that epidemic patterns over the field scale did not generally satisfy the Complete Randomness Hypothesis (CRS) but rather showed either sparse or aggregated infection points, the former being typical of the initial epidemic stages and the latter being typical of the epidemic exponential growth phase. A moderate spatial preferential patter caused by row effect was found from multidirectional and bidirectional two-dimensional autocorrelation and cross-correlation functions. Probability distributions of direction and distance of newly infected and recovered plants relative to existing infected plants were generally similar and highlighted no preferential direction of epidemic progression and regression with the exception of the direction of rows in the vineyard setting, and distances below 3 m in the majority of cases, which were in line with typically short-distance mobility by the vector. Finally, none of the recovered plants showed symptoms of infection after recovery over the time frame of these analyses. Overall, the results of this work suggest that both primary (from outside the vineyard) and secondary (within vineyard) infections contribute to disease spread. Therefore, an integrated control management of the disease aimed at removing sources of infection outside the vineyard (gone-wild *Vitis* plants that can be infected by FD and host the vector) and inside the vineyard (infected cultivated grapevines) is needed. The removal of infected plant in the vineyard or, at least, a prompt removal of the symptomatic shoots, is highly advisable to prevent secondary spread of FD within vineyard. Although our work did not investigate the influence of *S. titanus* population on disease spread, it is clear that suppression of vector population by insecticide applications is compulsory to minimize the spread of the disease that, in the absence of vector control, can rapidly affect the whole vineyard.

## Author Contributions

FM has contributed with the analyses, DB has contributed with analyses and data collection, LG, SP, and CM have contributed with field data collection. All authors have contributed to manuscript writing.

## Conflict of Interest Statement

The authors declare that the research was conducted in the absence of any commercial or financial relationships that could be construed as a potential conflict of interest.

## List of Symbols:

*d*: [L] distance between plants
*x, y*: [L] spatial coordinates
*M_i_*: [-] survey map of *i*-type plants
*N*: [-] total number of plants
*P_i_*: [-] total number of *i*-type plants
*t*: [T] time
*z*: [-] standard normal variable
*_t_*: subscript for symptomatic plants
*_nt_*: subscript for newly symptomatic plants
*_d_*: subscript for dead plants
*_r_*: subscript for recovered plants
*_c_*: subscript for removed plants
